# Porous Tantalum Coatings Prepared by Vacuum Plasma Spraying Enhance BMSCs Osteogenic Differentiation and Bone Regeneration *In Vitro* and *In Vivo*

**DOI:** 10.1371/journal.pone.0066263

**Published:** 2013-06-11

**Authors:** Ze Tang, Youtao Xie, Fei Yang, Yan Huang, Chuandong Wang, Kerong Dai, Xuebin Zheng, Xiaoling Zhang

**Affiliations:** 1 The Key Laboratory of Stem Cell Biology, Institute of Health Sciences, Shanghai Institutes for Biological Sciences (SIBS), Chinese Academy of Sciences (CAS) and Shanghai Jiao Tong University School of Medicine (SJTUSM), Shanghai, P.R. China; 2 Shanghai Key Laboratory of Orthopaedic Implant, Department of Orthopaedic Surgery, Shanghai Ninth People’s Hospital, Shanghai Jiao Tong University School of Medicine (SJTUSM), Shanghai, P.R. China; 3 Shanghai Institute of Ceramics, Chinese Academy of Sciences, Shanghai, P.R. China; Leibniz Institute of Age Research - Fritz Lipmann Institute, Germany

## Abstract

Tantalum, as a potential metallic implant biomaterial, is attracting more and more attention because of its excellent anticorrosion and biocompatibility. However, its significantly high elastic modulus and large mechanical incompatibility with bone tissue make it unsuitable for load-bearing implants. In this study, porous tantalum coatings were first successfully fabricated on titanium substrates by vacuum plasma spraying (VPS), which would exert the excellent biocompatibility of tantalum and alleviate the elastic modulus of tantalum for bone tissue. We evaluated cytocompatibility and osteogenesis activity of the porous tantalum coatings using human bone marrow stromal cells (hBMSCs) and its ability to repair rabbit femur bone defects. The morphology and actin cytoskeletons of hBMSCs were observed via electron microscopy and confocal, and the cell viability, proliferation and osteogenic differentiation potential of hBMSCs were examined quantitatively by PrestoBlue assay, Ki67 immunofluorescence assay, real-time PCR technology and ALP staining. For in vivo detection, the repaired femur were evaluated by histomorphology and double fluorescence labeling 3 months postoperation. Porous tantalum coating surfaces promoted hBMSCs adhesion, proliferation, osteogenesis activity and had better osseointegration and faster new bone formation rate than titanium coating control. Our observation suggested that the porous tantalum coatings had good biocompatibility and could enhance osseoinductivity in vitro and promote new bone formation in vivo. The porous tantalum coatings prepared by VPS is a promising strategy for bone regeneration.

## Introduction

In the last few years, great interest has been focused on tissue engineering as a potential therapeutic approach for musculoskeletal diseases. The role of metallic implants either for osteo-synthesis or for arthroplasty has been tested in preclinical and clinical settings. An ideal implant material should have appropriate elastic modulus, corrosion resistance, good biocompatibility and favor bone anchorage. However, most medical implant materials do not simultaneously fulfill all these characteristics. Accordingly, various coatings have been developed to improve the biocompatibility and osseoinductivity of load-bearing materials. Hydroxyapatite (HA) coating is one of outstanding examples of such coatings. HA-coated titanium (Ti) implants have the good biological activity of HA and the excellent mechanical properties of Ti alloy, and are thus widely used in orthopedic surgery as bone replacement materials [1]. Unfortunately, HA and other ceramic coatings are brittle and have the problem of “debond” from load-bearing substrate materials [2], [3], which hinder their extensive application. Thus, new type of coatings with good biocompatibility, osseoinductivity, long-term chemical stability, and firm bonding with substrate are still demanded for medical implants.

Tantalum (Ta) may be a potential coating material for medical implants. Ta (atomic number 73) is a rare transition metal that is highly corrosion resistant and inert in vivo [4]. It has been used in medical practice since the mid-1900s, and shows good medical biocompatibility and safety [2], [5]. Ta is considered as a potential biomaterial given its excellent chemical stability, body fluid resistance, biocompatibility, and biologic fixation with bone tissues [6]–[13]. However, its significantly high elastic modulus and large mechanical incompatibility with bone tissue make it unsuitable as bulk medical implant. Creating a porous layer on implant surfaces may enable the medical applications of Ta. Trabecular Metal™ is one such open cell porous Ta marketed by Zimmer Inc. (Warsaw, IN, USA) [14], [15]. These porous Ta components offer a low modulus of elasticity, high surface frictional characteristics, and excellent osseointegration properties (i.e., bioactivity, biocompatibility, and in-growth properties) [6], [13]. However, the relatively high cost and difficulty in fabrication (i.e., processing in an inert atmosphere, non-solderability, grinding difficulty, and high melting temperature) of Ta restrict its widespread use in medical practice [11]. In this study, vacuum plasma spraying (VPS) was first used to fabricate porous Ta coatings on titanium (Ti) substrates. The VPS technology is an economical method for fabricating porous coatings at very high melting temperatures. The porous structure of Ta coatings can effectively alleviate the mechanical incompatibility between Ta implants and host bone tissues. Thus, porous Ta-coated Ti implants, which combine the excellent biocompatibility of Ta and the good mechanical properties of Ti, can be used in medical implants.

Bone repair requires a cellular source with the ability of differentiation into bone together with a scaffold that allows the adhesion and proferation of these cells. Bone marrow mesenchymal stem cells (BMSCs) research is currently an exciting area of interest, since these cells have the ability to differentiate into several cell types, including osteoblasts, chondrocytes and adipocytes. In this regard, they have been extensively assessed for bone defects treatment [16]. This study aimed to investigate the biocompatibility, osteogenesis, and osseointegration of Ta coatings applied by VPS. Human bone marrow stromal cells (hBMSCs) were used for in vitro cellular adhesion, proliferation, and osteogenetic differentiation evaluation. Rabbit femur implant models were used to evaluate the osseointegration and new bone formation ability of porous Ta coating in vivo. This would be of great impact to support its application for clinical purposes, especially in the bone reconstructive techniques.

## Materials and Methods

### Ethics Statement

hBMSCs (Human bone marrow stromal cells) were donated by patients with written informed consent and this experiment was approved by Independent Ethics Committee of Shanghai Ninth People’s Hospital affiliated to Shanghai Jiao Tong University School of Medicine (SJTUSM). The rabbits were obtained from the Laboratory Animal Center of Shanghai Ninth People’s Hospital affiliated to Shanghai Jiao Tong University School of Medicine (SJTUSM) (Certificate number SCXK 2007-0007). Handling of the animals was in accordance with policies of Shanghai Ninth People’s Hospital affiliated to Shanghai Jiao Tong University School of Medicine (SJTUSM) and approved by the Animal Experimental Ethics Committee, Shanghai Ninth People’s Hospital affiliated to Shanghai Jiao Tong University School of Medicine (SJTUSM) (Permit Number: HKDL [2012]5).

### Material Preparation

Ta and Ti coatings were prepared by a VPS system (Sulzer, Winterthur, Switzerland). Medical Ti-6Al-4V substrate was used to prepare Ta-coated implants and interface samples. The samples for investigating hBMSC proliferation and differentiation in vitro were cuboid with dimensions of 10 mm×10 mm×2 mm and cylindrical with dimensions of Ø 33 mm×2 mm. The samples for implantation in vivo to examine osseointegration and new bone formation rate were cylindrical with dimensions of Ø 2 mm×10 mm.

### Surface Morphology

The coating surface was characterized using the following equipment: an X-ray diffraction (XRD) instrument (D/max 2550v, Rigaku, Japan; MDI Jade 6.5), a contact angle analyzer (SL200, Kino, Shanghai, P.R. China; with distilled water), a field emission scanning electron microscopy (SEM) system (JSM 6700F, JEOL, Akishima, Tokyo, Japan), and an electron probe microanalyzer (EPMA, JXA-8100, JEOL, Akishima, Tokyo, Japan).

### Isolation and Culture of hBMSCs

The hBMSCs were isolated and cultured as previously described [17]. A humidified 37°C/5% CO_2_ incubator (MCO-18AIC (UV), SAVYO, Panasonic, Kadoma, Osaka, Japan) was used for cell culture with *growth medium* [α-MEM (Hyclone, Thermo Fisher Scientific, Waltham, MA, USA) supplemented with 10% FBS (Hyclone, Thermo Fisher Scientific, Waltham, MA, USA), 100 U/mL penicillin, and 100 mg/L streptomycin (Hyclone, Thermo Fisher Scientific, Waltham, MA, USA)].

### Osteogenic Induction

The hBMSCs were seeded in six-well plates (Corning, Corning, NY, USA) at a density of 5×10^4^ cells/well. After confluence in *growth medium*, the cells were treated with *osteogenic induction medium* [α-MEM supplemented with 10% FBS, 50 µM l-ascorbic acid 2-phosphate (Sigma–Aldrich, St. Louis, MO, USA), 10 mM β-glycerophosphate (Sigma–Aldrich, St. Louis, MO, USA), 100 nM dexamethasone (Sigma–Aldrich, St. Louis, MO, USA), 100 U/mL penicillin, and 100 mg/L streptomycin], and then replaced every 3 days. After 7 days, the cells were stained using a BCIP/NBT ALP Color Development Kit (Beyotime, P.R. China). Cells cultured in normal growth medium served as control.

### Adipogenic Induction

The hBMSCs were seeded in six-well plates (Corning, Corning, NY, USA) at a density of 2×10^5^ cells/well. After confluence in *growth medium*, the cells were treated with *adipogenic induction medium* [α-MEM supplemented with 10% FBS, 1 µM dexamethasone phosphate, 200 µM Indomethin (Sigma–Aldrich, St. Louis, MO, USA), 10 µg/mL insulin (Sigma–Aldrich, St. Louis, MO, USA), 0.5 mM 3-isobutyl-1-methylxanthine (IBMX, Sigma–Aldrich, St. Louis, MO, USA), 100 U/mL penicillin, and 100 mg/L streptomycin] for 2 days. Thereafter, *adipogenic maintenance medium* [(α-MEM supplemented with 10% FBS, 10 µg/mL insulin, 100 U/mL penicillin, and 100 mg/L streptomycin)] was used and replaced every day for 14 days. Oil Red O staining was performed and then the sample was observed under a microscope (Nikon, Shinjuku, Tokyo, Japan). Cells cultured in normal growth medium served as control.

### Cell Proliferation and Adhesion

The 10 mm×10 mm×2 mm samples were placed in 24-well plates (Corning, Corning, NY, USA) and the hBMSCs were seeded at 2×10^4^ cells/well with the *growth medium*. After 24 h, the samples were transferred to new 24-well plates for further experiment. DRAQ5 (Danvers, MA, USA) was used as fluorescent DNA dye. The samples were scanned with an Odyssey near-infrared scanner (LI-COR Biosciences, Millipore, Billerica, MA, USA) to determine the proliferation rate variation of hBMSCs on porous Ta and Ti coating. PrestoBlue Cell Viability Reagent (Invitrogen, Life Technologies, Carlsbad, CA, USA) was used according to the manufacturer’s instructions to test cell viability, and the absorbance was obtained using a microplate reader (Infinite M200 pro, TECAN, Switzerland) at wavelengths of 570 and 650 nm. LIVE/DEAD Cell Viability Assays (Invitrogen, Life Technologies, Carlsbad, CA, USA) were performed as described in the manual. F-actin was identified by fluorescent phallotoxin (Phalloidin-TRITC, Sigma–Aldrich, St. Louis, MO, USA). Anti-Ki67 antibody (Abcam, Cambridge, UK) was used to detect Ki-67 protein expression in hBMSCs, and DAPI (Sigma–Aldrich, St. Louis, MO, USA) was used to indicate the nucleus of hBMSCs. LIVE/DEAD Cell Viability Assays, fluorescent phallotoxin, and anti-Ki67 antibody/DAPI were observed by confocal laser scanning microscopy (LSM510, Carl Zeiss AG, Oberkochen, Germany).

The CFSE labeling experiment was carried out as described [18], [19]. CFDA-SE (Sigma–Aldrich, St. Louis, MO, USA) was used for labeling. The CFSE labeled hBMSCs were seeded on the Ø 33 mm×2 mm samples which placed in six-well plates (Corning, Corning, NY, USA). After 24 h, the samples were transferred to new six-well plates. The hBMSCs with CFSE labeling were cultured for total 7 days and then analyzed by flow cytometers FACS Aria (Becton Dickinson, Franklin Lakes, NJ, USA).

### ALP Staining of hBMSCs on Coatings

The Ø32 mm×2 mm samples with coating were placed in six-well plates (Corning, Corning, NY, USA) and the hBMSCs were seeded at 5×10^4^ cells/well with the *growth medium*. After 24 h, the samples were transferred to new six-well plates, and after another 24 h, the growth medium was replaced with the *osteogenic induction medium*, which was replaced every 3 days. The cells were stained using a BCIP/NBT ALP Color Development Kit (Beyotime, P.R. China), and then scanned with an Odyssey near-infrared scanner (LI-COR Biosciences, Millipore, Billerica, MA, USA) and a normal scanner (Cannon, Tokyo, Japan).

### In-Cell Western

In-Cell Western assay was performed following the protocol of CST Ltd. to evaluate the protein expression of ALP in hBMSCs on porous Ta coatings. Paraformaldehyde (4%) was used for fixation. Odyssey Blocking Buffer (LI-COR Biosciences, Millipore, Billerica, MA, USA) was used as a blocking and antibody-diluting buffer. ALP antibody (Santa Cruz Biotechnology, Santa Cruz, CA, USA) was used to detect the protein expression of ALP in hBMSCs on the coatings. After incubation, Infra-Red Secondary Antibody IRDye (Rockland Immunochemicals, Gilbertsville, PA, USA) was used as a secondary antibody and DNA staining dye DRAQ5 (Danvers, MA, USA) was used as normalizing agent [20]. The result was acquired by an Odyssey Infrared Imaging System (LI-COR Biosciences, Millipore, Billerica, MA, USA).

### Real-time RT-PCR

Total RNA of cells was isolated using TriPure Isolation Reagent (Roche, Basel, Switzerland) according to the manufacturer’s instructions. Equivalent amount of RNA samples were reverse transcribed for first-strand cDNA using a RevertAid First Strand cDNA Synthesis Kit (Fermentas, Thermo Fisher Scientific, Waltham, MA, USA). Real-time PCR was performed by Lightcycler480 (Roche, Basel, Switzerland) using SYBR Premix Ex TaqTM (Takara, Otsu, Shiga, Japan) according to the manufacturer’s instructions. GAPDH was used as a reference. The real-time PCR conditions were as follows: denaturation at 95°C for 10 s, 40 cycles at 95°C for 10 s, and 60°C for 20 s. Dissociation was performed for a melting curve analysis to monitor and avoid non-specific amplification as well as primer dimers. The collected data were analyzed by the advanced relative quantification method using Roche Lightcycler480 software.

### Animals and Implantation

New Zealand white rabbits (male, 3 months old, 2.8–3.0 kg) were used as the femur implant model. The operation was performed under aseptic conditions by chloral hydrate intramuscular injection. Defect in each femoral condyle was made by a Ø 2 mm drill toward the medial epicondyle orientated perpendicular to the longitudinal and sagittal axes [17]. After placing the implant in the hole, incisions were closed with sutures. To avoid wound infection, each animal was given an intramuscular injection of 400 000 U penicillin per day for 3 days after operation.

### Double Fluorescence Labeling and Harvesting of Bone Samples

The first labeling was performed by intravenous injection 10 days before sacrificing the rabbits, and the second labeling was performed 3 days before sacrificing. Calcein (8 mg/kg, Sigma–Aldrich, St. Louis, MO, USA) was used for both labelings.

### Histomorphology

After cleaning the soft tissue, the samples were fixed in 4% paraformaldehyde buffer in PBS for 10 days and then dehydrated in successive alcohol concentrations (70%, 75%, 80%, 85%, 90%, 95%, and 100% per day). The dehydrated sample was embedded in Technovit 7200VLC (Exakt, Norderstedt, Hamburg, Germany) for 2 days using the EXAKT 510 Dehydration and Infiltration System (Exakt, Norderstedt, Hamburg, Germany), and then polymerized by the EXAKT 520 Light Polymerization System (Exakt, Norderstedt, Hamburg, Germany). Subsequently, 300 µm sections were cut perpendicular to the implants using Saw Microtome Leica SP1600 (Leica, Wetzlar, Germany), ground to 50 µm by the EXAKT 400 CS Micro Grinding System with AW 110 controller (Exakt, Norderstedt, Hamburg, Germany), and finally stained by Van Gieson’s Picric–Fuchsin stain for histomorphometry.

### Histomorphometry

Different histomorphometric parameters were obtained using a microscope (LEICA DM 4000B, German). A semiautomatic image analysis system (BIOQUANT) was used to measure the surfaces of the sections. The area of 50 pixels around the implant was selected as the region of interest (ROI), amounts of bone tissues in the ROI were calculated by using the measurement tools. Bone volume fractions (ratio of bone tissue area to the total area in the ROI) of different samples were calculated based on Van Gieson’s Picric–Fuchsin staining and compared statistically.

### Statistical Analysis

Statistical analysis was performed using SAS JMP (Cary, NC, USA). Aspin-Welch-Satterthwaite-Student's t-test (t-test works even if variances in the two sample groups are different) were used to compare the means of the two sample groups.

## Results

### Identification of hBMSCs

**Figure 1:**
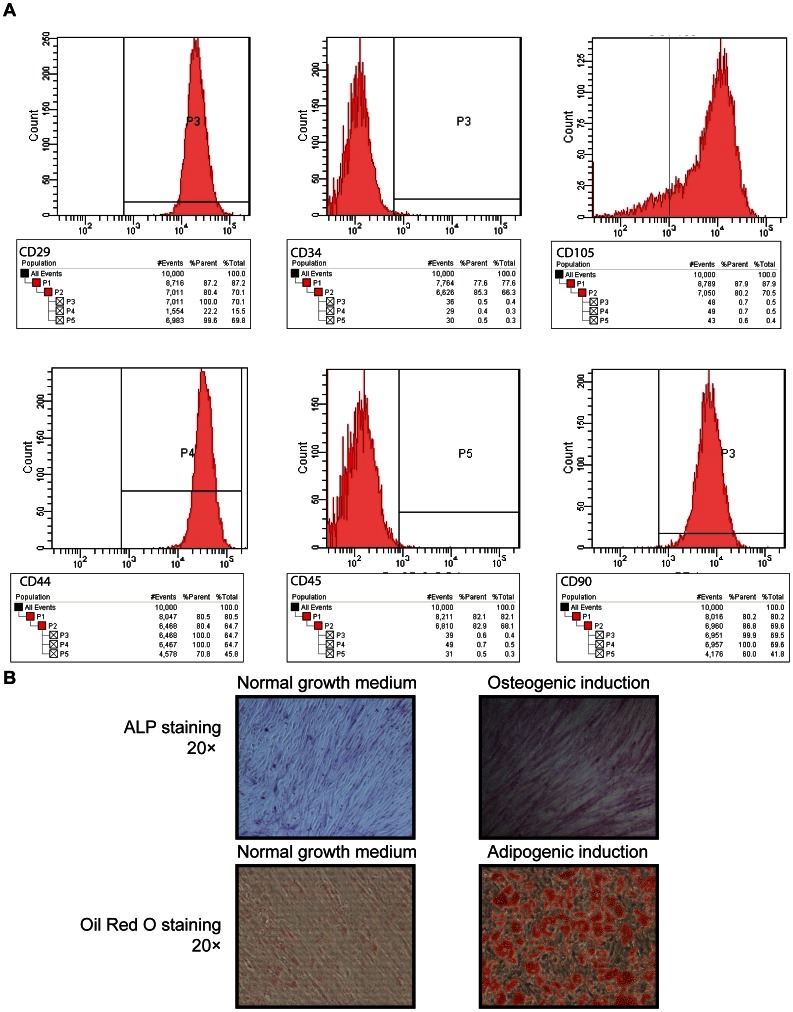
Identification and lineage differentiation potential of hBMSCs. (**A**) hBMSC identification with the surface markers CD29^+^, CD34^−^, CD105^+^, CD44^+^, CD45^−^, and CD90^+^. (**B**) The hBMSC lineage differentiation: osteoblasts (ALP stain), and adipocytes (Oil Red O stain).

The hBMSCs were isolated as previously described [17], and then used to evaluate the biocompatibility and osteoinductivity of porous Ta and Ti coatings. The isolated cells expressed the MSC markers CD29, CD105, CD44 and CD90, but were negative for the hematopoietic marker CD34 and leukocyte marker CD45 (**Figure 1A**). These findings were similar to the hBMSC population phenotypic characteristics described in previous research [21]–[23]. The differentiation potential of hBMSCs was also investigated by osteogenic and adipogenic induction, followed by ALP and Oil Red O staining. The results demonstrated the hBMSCs’ potential to differentiate into the lineages of osteoblast and adipocyte (**Figure 1B**).

### Surface Morphology of Coatings

**Figure 2:**
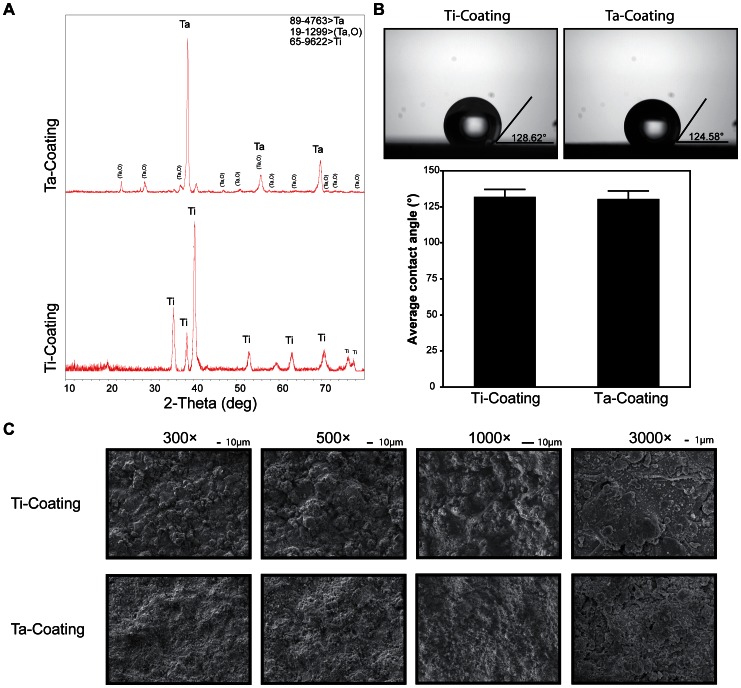
XRD and surface morphology identification of Ta and Ti coatings. (**A**) XRD patterns (**B**) contact angle of porous Ta and Ti coatings (t-test, assuming unequal variances, error bars represent ± SD, *n* = 3; Ti coating, 131.917±5.10882; Ta coating, 130.455±5.55294; Prob>|t|, 0.7125 means no significant difference). (**C**) SEM images of the initial surface morphology of both coatings at 300×, 500×, 1000×, and 3000× magnifications.

The XRD results showed that the Ti coating surface was mainly composed of Ti, and the composition of the Ta coating was major Ta and a minor Ta oxide (**Figure 2A** **)**. The oxide layer of Ta (Ta_2_O_5_) formed on the surface (**Figure 2A**) was quite stable at various pH values [2], [5]. The contact angle of porous Ta coating surface insignificantly differed from that of the Ti coating (**Figure 2B**) (*n* = 3; Ti coating, 131.917±5.10882; Ta coating, 130.455±5.55294; P = 0.7125). The initial surface morphology of Ta and Ti coatings was observed by SME at 300×, 500×, 1000×, and 3000× magnifications (**Figure 2C** **)**, showing that the surface roughness of both Ta and Ti coatings was similar.

### Cell Shape and Cytoskeletal Tension of hBMSCs on Ta Coatings

**Figure 3:**
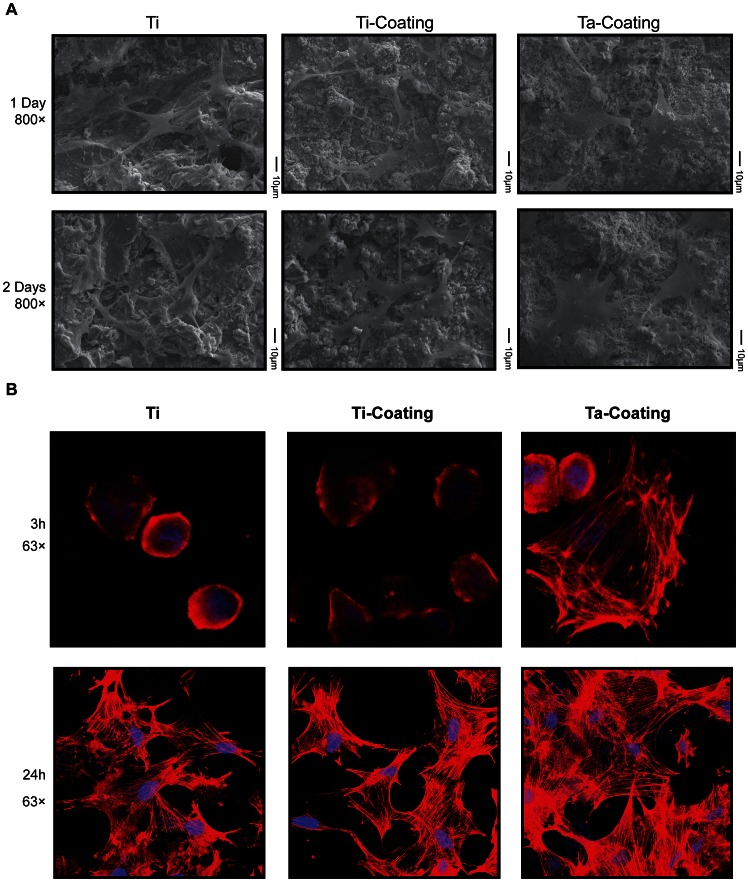
Morphology of hBMSCs cultured on Ta and Ti coatings. (**A**) SEM images of the surface morphology of both coatings with cultured hBMSCs at 800× magnifications. (**B**) Confocal laser scanning microscopy images of single hBMSC F-actin cytoskeletal morphology on both coatings for 3 and 24 h. (red, phalloidin for F-actin; blue, DAPI for nucleus).

The hBMSCs displayed much better expanding on the porous Ta coating surface compared with the Ti coating control and the uncoated control (**Figure 3A and B**). In **Figure 3A** **,** the SME image showed that the hBMSCs on Ta coating had a flattened expanded shape. F-actin cytoskeletal staining (**Figure 3B** **)** indicated the different cytoskeletal tensions and the flattened expanded shape of hBMSCs on Ta coating compared with the contracted shape on Ti coating.

### Adhesion and Proliferation of hBMSCs on Ta Coatings

**Figure 4:**
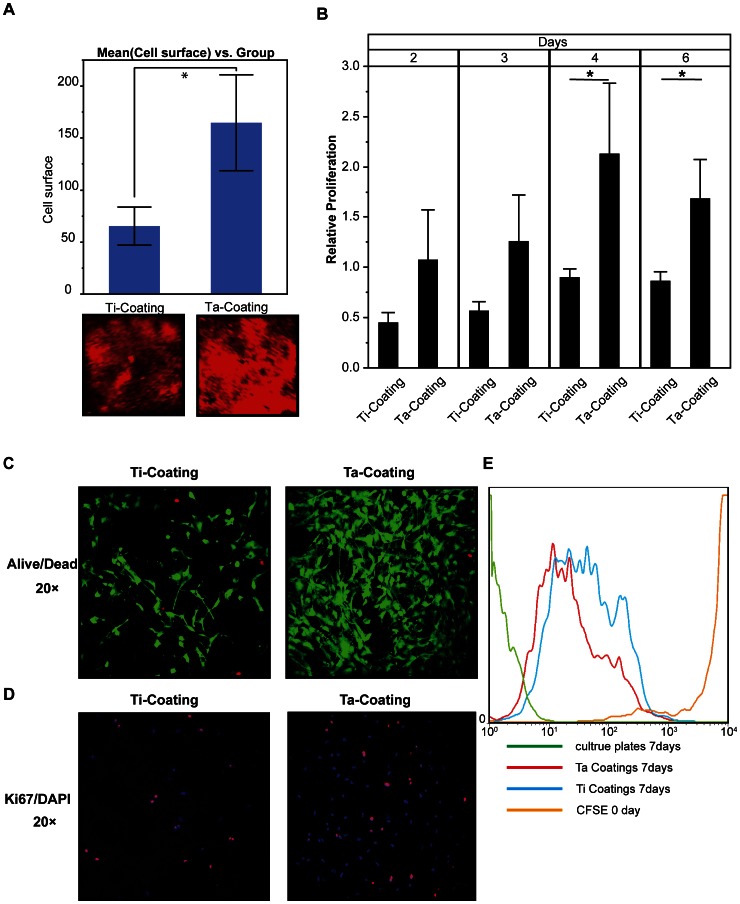
hBMSC viability and proliferation on the surface of Ta and Ti coatings. (**A**) Quantification of Odyssey scanning of hBMSCs on the surface of Ta and Ti coatings stained with DRAQ5 in 48 h (t-test, assuming unequal variances, error bars represent ± SD, *n* = 3; Ti coating, 65.377±18.2972; Ta coating, 164.80±46.1212; Prob>|t|, 0.0498*). (**B**) Cell viability of hBMSCs on both coatings measured by PrestoBlue assay (t-test; error bars represent ± SD, *n* = 3; day 2; Ti coating, 0.45268±0.097954; Ta coating, 1.0767±0.495686; Prob>|t|, 0.992; day 3; Ti coating, 0.56950±0.089554; Ta coating, 1.26228±0.458399; Prob>|t|, 0.0620; day 4; Ti coating, 0.90275±0.082739; Ta coating, 2.13239±0.699593; Prob>|t|, 0.0390*; day 6; Ti coating, 0.86752±0.089092; Ta coating, 1.68688±0.386935; Prob>|t|, 0.0233*; the cell viability of hBMSCs on normal cell culture plate serviced as calibrator). (**C**) LIVE/DEAD Cell Viability Assays of hBMSCs on the surface of the coatings in 48 h. (**D**) Ki67 and DAPI stains of hBMSCs on the surface of the coatings in 48 h. (**E**) The flow cytometric analyses of hBMSC with CFSE on the surface of the coatings on day 7.

An immunofluorescence experiment was conducted in which the DNA dye Draq5 was applied to monitor cell number (density). The result (**Figure 4A**) showed that the hBMSCs proliferated much faster on porous Ta than on Ti coating in 48 h (*n* = 3; Ti coating, 65.377±18.2972; Ta coating, 164.80±46.1212; P<0.0498*). In **Figure 4B**, the data of PrestoBlue assay showed that the hBMSCs had a significantly faster proliferation on the porous Ta coating surface than on the Ti coating control after 4 and 6 days (*n* = 3). For day 2, the data were as follows: Ti coating, 0.45268±0.097954; Ta coating, 1.0767±0.495686; P<0.992. For day 3, the data were as follows: Ti coating, 0.56950±0.089554; Ta coating, 1.26228±0.458399; P<0.0620. For day 4, the data were as follows: Ti coating, 0.90275±0.082739; Ta coating, 2.13239±0.699593; P<0.0390*. For day 6, the data were as follows: Ti coating, 0.86752±0.089092; Ta coating 1.68688±0.386935; P<0.0233*. LIVE/DEAD Cell Viability assay and anti-Ki67 immunofluorescence assay (**Figures 4C and 4D** **,** respectively) demonstrated not only faster proliferation but also less cell death of hBMSCs on porous Ta coating than on Ti coating in 48 h. The CFSE labeling results (**Figure 4E**) also showed hBMSCs on Ta coating surface had a higher proliferation rate than those on Ti coating surface.

### ALP and RUNX2 Expression in hBMSCs on Ta Coatings

**Figure 5:**
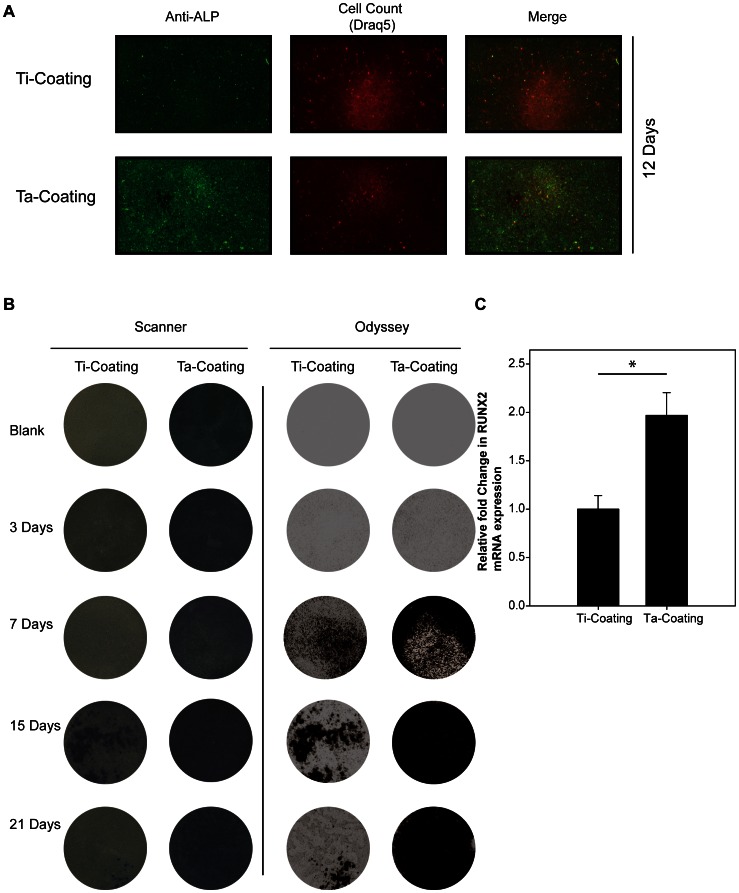
ALP and RUNX2 expression of hBMSCs on Ta and Ti coatings with osteogenic induction. (**A**) Odyssey scanning of the ALP protein expression in hBMSCs on the coatings with DRAQ5 as reference, 12 days. (**B**) ALP activity stain assay of hBMSCs on the coatings in 3, 7, 15, and 21 days with a white light scanner and near-infrared Odyssey scanner. (**C**) Real-time PCR detection of the mRNA expression of RUNX2 gene in hBMSCs on the coatings with osteogenic induction for 21 days. GAPDH was used as reference gene (t-test, assuming unequal variances, *n* = 3; Ti coating, 1±0.1404; Ta coating, 1.9678±0.2370; Prob>|t|, <0.01*).

The hBMSCs were cultured on porous Ta and Ti coatings with the osteogenic medium, and then ALP and RUNX2 were detected as osteoplastic differentiation-related markers. Higher ALP activity and expression were detected (**Figures 5A and 5B**) on porous Ta coating by immunofluorescence detection of ALP with DRAQ5 as reference (12 days, 0.057 vs 0.029 with Odyssey calculation) and ALP staining (7, 15, and 21 days). Real-time RT-PCR was used to detect the mRNA expression of RUNX2 when the hBMSCs were cultured on the porous Ta coating and on the porous Ti coating control in osteogenic medium after 21 days. The expression of RUNX2 was significantly enhanced in hBMSCs cultured on porous Ta coating compared with Ti control coating (**Figure 5C**) (*n* = 3; Ti coating, 1±0.1404; Ta coating, 1.9678±0.2370; P<0.01*). These results suggested that the porous Ta coating may be more beneficial for hBMSC osteogenesis than Ti coating.

### Osseointegration of Implants with Porous Ta Coating in vivo

**Figure 6:**
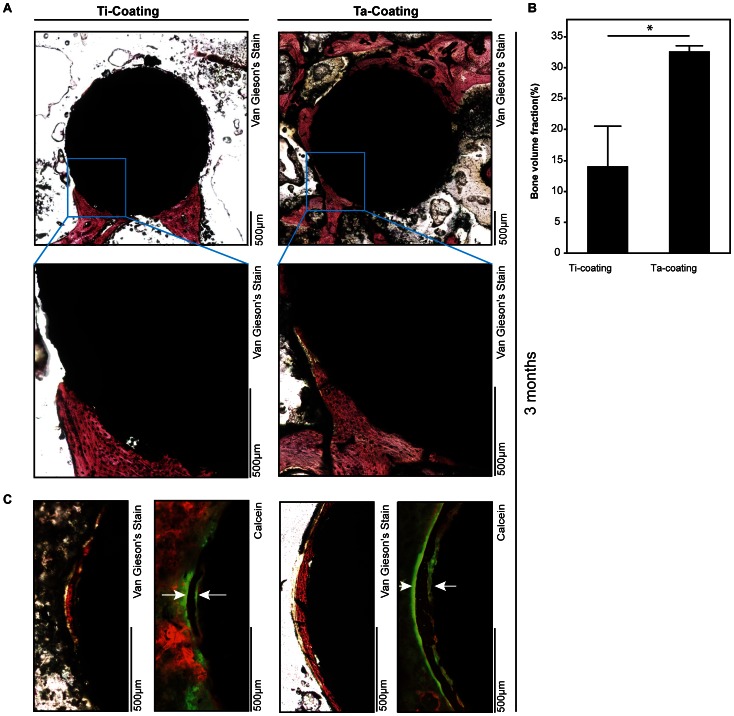
New bone formation around porous Ta and Ti coating implants in vivo. (**A**) Van Gieson’s Picric–Fuchsin stain of new bone formation around implants. Red indicates the bone around implants in 3 months. (**B**) Quantification of new bone formation around the coating implants in 3 months in vivo (t-test, assuming unequal variances, error bars represent ± SD, *n* = 3; Ti coating, 14.0741±6.46293; Ta coating, 32.6501±0.90721; Prob>|t|, 0.0358*). (**C**) Double fluorescence labeling was used to represent the mineral apposition rate of new bone formation. Green with white arrow indicates the calcein fluorescence labels in 3 months.

Van Gieson’s Picric–Fuchsin stain of transverse sections was used to show the osseointegration of the porous Ta and Ti coatings. The mineral apposition rate of new bone formation was also investigated by measuring the distances of double fluorescence labeling. After implantation for 3 months, histomorphometric parameters clearly indicated that more new bone was formed around the porous Ta coating implant than around the Ti coating implant (**Figures 6A and 6B**) (*n* = 3; Ti coating, 14.0741±6.46293; Ta coating, 32.6501±0.90721; *P*<0.0358*). The double fluorescence labeling (**Figure 6C**) also showed that the mineral apposition rate of new bone formation was higher in the group implanted with porous Ta coating.

## Discussion

Over the last two decades, a variety of porous coatings and materials have been used to achieve biological fixation of implants. Ti alloys are found to be very suitable materials for load-bearing in bioimplant applications because of their good and reliable mechanical properties. Unfortunately, like most metals, Ti exhibits poor osteoinductive properties. Among metallic biomaterials, Ta is gaining increased attention as a new biomaterial. Ta has been demonstrated to be corrosion resistant and bioactive in vivo. Although Ta has been used for plates and suture wires in orthopedic, craniofacial surgery, and dentistry [24], the relatively high manufacturing cost and difficulty in fabrication have limited its widespread acceptance. To utilize fully the biocompatibility of Ta for load-bearing metal implants, in this work, porous Ta coating were prepared with Ti as substrate. The biocompatibility and osteoinductivity of Ta coatings applied by VPS were then examined.

The in vitro hBMSC-material interaction results clearly indicated that the porous Ta coating surface significantly improved the adhesion and proliferation of hBMSCs, as assessed by SEM cell morphology, F-actin cytoskeletal staining, immunofluorescence, and PrestoBlue assay. The characteristics and biocompatibility of implant surface material were closely related to cell-material interactions, and can influence cell response and behavior [25], [26]. Our results showed the superior cell adhesion and proliferation, as well as less cell death of hBMSCs on the porous Ta coating compared with Ti coating. These findings indicated that Ta surfaces were biocompatible and significantly influenced hBMSC biological behavior. Generally, the initial adhesion influences the subsequent differentiation of stem cells [3], [27]–[36]. The different cytoskeleton signals from adhesion are transduced to the nucleus, where they can result in a change in gene expression and, subsequently, differentiation [36], [37]. McBeath et al. [38] proved that the cell shape regulates hBMSCs’ commitment to osteoblast or adipocyte fate (i.e., hBMSCs allowed to adhere, flatten, and expanded undergo osteogenesis, whereas condensed, round cells become adipocytes), and that F-actin cytoskeletal is involved in this process. In this study, F-actin cytoskeletal staining reveals the flattened expanded shape of hBMSCs on Ta coating compared with the contracted shape on Ti coating. Therefore, the difference in hBMSCs’ F-actin cytoskeleton on porous Ta and Ti coatings partly explained the higher osteogenesis of hBMCs on the porous Ta coating implants.

Moreover, the porous Ta coating not only enhanced hBMSC adhesion and proliferation, but also stimulated mesenchymal stem cell osteogenetic differentiation in vitro compared with porous Ti. This finding was confirmed by the enhanced ALP and RUNX2 activity on Ta coating. The possible explanation was that the bone-like elasticity substrates may have been better for bone regeneration and contributed to the differentiation lineage of the hBMSCs [39], [40]. Thus, given its similar elasticity to cancellous bone, porous Ta coating was more beneficial for osteogenesis. In vivo, which used a rabbit femur implant model, we found that the implants coated with Ta had a higher rate of osseointegration than the Ti coating implants because of the excellent biocompatibility and bioactivity of Ta. These key properties of porous Ta can be attributed to its ability to form a self-passivating surface oxide layer. This surface layer leads to the formation of a bone-like apatite coating in vivo, ensuring excellent bone and fibrous in-growth properties for rapid and substantial bone–soft tissue attachment [6]. Our in vivo results on the accelerated rate of new bone formation on Ta coating implant compared with Ti coating implant well agreed with in vitro studies demonstrating enhanced stem cell adhesion, proliferation, and osteogenic differentiation on a Ta coating surface over a Ti coating surface.

These results revealed the ability of porous Ta coating to support the differentiation of mesenchymal stem cells into osteoblasts in vitro and of matured bone cells in vivo. Porous Ta was found to have excellent biocompatibility and be safe to use in vivo [2]. Core decompression with porous Ta implants has shown encouraging success rates in early clinical results among patients with advanced stage osteonecrosis [9]. Thus, porous Ta is indeed an attractive option for clinical applications. Although long-term experimental and clinical studies are required to verify the advantages and outcomes of such an implant, a novel application of porous Ta as an orthopedic implant coating applied by VPS for promoting bone regeneration was presented.

### Conclusion

Porous Ta coatings were fabricated by VPS with Ti alloy as substrate. The deposition of porous Ta layer effectively alleviated the mechanical incompatibility between metal implant and bone tissue. The biocompatibility, osteoinductivity, and osseointegration of the Ta coated implants were studied to determine their feasibility in clinical applications. In vitro, hBMSCs were used and the results showed that hBMSC adhesion, proliferation, and osteogenic differentiation were enhanced on the Ta coating surface compared with the Ti coating control. Moreover, in vivo implantation in a rabbit femur defect model confirmed the excellent osseointegration and new bone formation of the porous Ta coated implants. These results suggested that porous Ta coating applied by VPS is a promising strategy for bone regeneration.
